# Add-On MEmaNtine to Dopamine Antagonism to Improve Negative Symptoms at First Psychosis- the AMEND Trial Protocol

**DOI:** 10.3389/fpsyt.2022.889572

**Published:** 2022-05-20

**Authors:** Katharina O. Sandström, Olga B. Baltzersen, Anouk Marsman, Cecilie K. Lemvigh, Vincent O. Boer, Kirsten B. Bojesen, Mette Ø. Nielsen, Henrik Lundell, Daban K. Sulaiman, Mikkel E. Sørensen, Birgitte Fagerlund, Adrienne C. Lahti, Warda T. Syeda, Christos Pantelis, Esben T. Petersen, Birte Y. Glenthøj, Hartwig R. Siebner, Bjørn H. Ebdrup

**Affiliations:** ^1^Center for Neuropsychiatric Schizophrenia Research (CNSR), Centre for Clinical Intervention and Neuropsychiatric Schizophrenia Research (CINS), Mental Health Centre Glostrup, Glostrup, Denmark; ^2^Danish Research Centre for Magnetic Resonance, Centre for Functional and Diagnostic Imaging and Research, Copenhagen University Hospital Amager and Hvidovre, Copenhagen, Denmark; ^3^Department of Clinical Medicine, Faculty of Health and Medical Sciences, University of Copenhagen, Copenhagen, Denmark; ^4^Department of Psychology, University of Copenhagen, Copenhagen, Denmark; ^5^Department of Psychiatry and Behavioral Neurobiology, University of Alabama at Birmingham, Birmingham, AL, United States; ^6^Department of Psychiatry, Melbourne Neuropsychiatry Centre, The University of Melbourne and Melbourne Health, Carlton South, VIC, Australia; ^7^Section for Magnetic Resonance, DTU Health Tech, Technical University of Denmark, Lyngby, Denmark; ^8^Department of Neurology, Copenhagen University Hospital Bispebjerg and Frederiksberg, Copenhagen, Denmark

**Keywords:** antipsychotic-naïve first-episode schizophrenia, memantine, neuromelanin (NM), amisulpride (AMS), magnetic resonace imaging (MRI), magnetic resonance spectrography (MRS), randomized controlled (clinical) trial, glutamate

## Abstract

**Background:**

Antipsychotic drugs are primarily efficacious in treating positive symptoms by blocking the dopamine D2 receptor, but they fail to substantially improve negative symptoms and cognitive deficits. The limited efficacy may be attributed to the fact that the pathophysiology of psychosis involves multiple neurotransmitter systems. In patients with chronic schizophrenia, memantine, a non-competitive glutamatergic NMDA receptor antagonist, shows promise for ameliorating negative symptoms and improving cognition. Yet, it is unknown how memantine modulates glutamate levels, and memantine has not been investigated in patients with first-episode psychosis.

**Aims:**

This investigator-initiated double-blinded randomized controlled trial is designed to (1) test the clinical effects on negative symptoms of add-on memantine to antipsychotic medication, and (2) neurobiologically characterize the responders to add-on memantine.

**Materials and Equipment:**

Antipsychotic-naïve patients with first-episode psychosis will be randomized to 12 weeks treatment with [amisulpride + memantine] or [amisulpride + placebo]. We aim for a minimum of 18 patients in each treatment arm to complete the trial. Brain mapping will be performed before and after 12 weeks focusing on glutamate and neuromelanin in predefined regions. Regional glutamate levels will be probed with proton magnetic resonance spectroscopy (MRS), while neuromelanin signal will be mapped with neuromelanin-sensitive magnetic resonance imaging (MRI). We will also perform structural and diffusion weighted, whole-brain MRI. MRS and MRI will be performed at an ultra-high field strength (7 Tesla). Alongside, participants undergo clinical and neuropsychological assessments. Twenty matched healthy controls will undergo similar baseline- and 12-week examinations, but without receiving treatment.

**Outcome Measures:**

The primary endpoint is negative symptom severity. Secondary outcomes comprise: (i) clinical endpoints related to cognition, psychotic symptoms, side effects, and (ii) neurobiological endpoints related to regional glutamate- and neuromelanin levels, and structural brain changes.

**Anticipated Results:**

We hypothesize that add-on memantine to amisulpride will be superior to amisulpride monotherapy in reducing negative symptoms, and that this effect will correlate with thalamic glutamate levels. Moreover, we anticipate that add-on memantine will restore regional white matter integrity and improve cognitive functioning.

**Perspectives:**

By combining two licensed, off-patent drugs, AMEND aims to optimize treatment of psychosis while investigating the memantine response. Alongside, AMEND will provide neurobiological insights to effects of dual receptor modulation, which may enable future stratification of patients with first-episode psychosis before initial antipsychotic treatment.

**Clinical Trial Registration:**

[ClinicalTrials.gov], identifier [NCT04789915].

## Introduction

Antipsychotic medication (AP) is primarily effective in treating positive psychotic symptoms such as delusions, hallucinations, and disorganized thinking, which are cardinal symptoms of schizophrenia. However, AP does not ameliorate the accompanying negative symptoms (e.g., anhedonia, avolition, and social withdrawal), and cognitive deficits, which are highly disabling and predictive of patients’ long-term prognosis (1–3). Persistent negative symptoms have also shown to be a critical predictor for future treatment resistance (4). A growing body of evidence support that a shortening of the interval between onset of psychosis and initiation of an efficient intervention lead to better long-term outcome (5–7).

The pathophysiology of psychosis is complex and involves multiple neurotransmitters, e.g., serotoninergic- (8), GABAergic, and glutamatergic (9) systems. Nevertheless, modulation (particularly antagonism) of striatal dopamine D2 receptors (D2R) remain the key common denominator of all licensed antipsychotics (10). Even so, around 30% of patients with psychosis display inadequate response to antidopaminergic treatment (11, 12). Mounting evidence from post- mortem-, brain imaging-, genetic-, and pharmacologic challenge studies indicate glutamatergic dysregulation, specifically of the N-methyl-D-aspartate receptor (NMDAR), as part of the pathophysiology of psychosis and schizophrenia (13–15). Through excitotoxicity, glutamate dysregulation may underlie structural degeneration, e.g., in hippocampus, which is a key finding in patients with schizophrenia (16–19).

Using 3T magnetic resonance spectroscopy (MRS), we recently reported increased thalamic glutamate levels in antipsychotic-naïve patients with first episode psychosis (FEP) compared to healthy controls (HC). Importantly, high thalamic glutamate levels predicted poor treatment response (9). Other studies have also associated glutamate levels with treatment outcome (20–22). Nevertheless, cross-sectional studies of glutamate levels between patients with FEP and HC have shown inconsistent results, (21, 23, 24) likely reflecting differences in glutamate levels across brain regions and in patients’ variable previous antipsychotic exposure, as well as the complexity of the disorder, with individual differences regarding the neurobiological findings Studies have also shown that thalamic glutamate levels are heritable, genetically related to psychosis (25), and that in individuals at ultra-high risk of psychosis (UHR), thalamic glutamate levels are related to attention and level of functioning (26, 27). Moreover, we previously demonstrated that thalamic and anterior cingulate cortex (ACC) levels of glutamate are associated with symptomology and cognition, specifically spatial working memory, set-shifting and sustained attention both in UHR and FEP (9, 26, 28, 29).

Cutting-edge 7T MRS can non-invasively determine brain metabolite levels of e.g., γ-aminobutyric acid (GABA), N-acetylaspartate, N-acetylaspartyl and importantly, 7T allows for discrimination of glutamate concentrations from glutathione and glutamine (22, 30, 31). Moreover, multiple voxels/brain regions can be investigated with 7T. In the context of glutamate and psychosis, the thalamus, ACC, hippocampus, dorsolateral prefrontal cortex (DLPFC), and basal ganglia are of particular interest, e.g., (17, 29, 32–34).

Regarding brain structure, the use of structural 7T sequences offers increased sensitivity to quantify neurodegenerative processes such as volume loss e.g., by hippocampal subfield segmentation (35).

Also, high-resolution quantification of tissue parameters such as iron deposition using quantitative susceptibility mapping allow characterization of subtle changes in the “dopaminergic regions” of the basal ganglia, including striatum, pallidum, and substantia nigra (SN) (35). Recently, a promising non-invasive method for *in vivo*-investigating dopamine in neuropsychiatric illness has been developed. Neuromelanin-sensitive MRI (NM-MRI) purports to detect the content of neuromelanin (NM), a product of dopamine metabolism that accumulates in the SN. NM may be regarded as a proxy of structural and functional integrity of dopaminergic cells in SN (36). Findings indicate that NM-MRI signals correlate with NM concentration, dopamine levels in the striatum and severity of psychosis in schizophrenia, even without neurodegeneration (37).

NMDA-receptor modulation has been investigated in several preclinical studies (38–40). Interestingly, different NMDA-receptor compounds lead to various effects on molecular, anatomical, and behavioral levels. Specifically, memantine exerts opposite effects than ketamine on the genetic level, which may underlie the two drugs’ different clinical effects (38, 41).

Against this background, modulation of glutamatergic NMDAR has been investigated as a potential treatment target in patients with schizophrenia or other psychotic disorders. Memantine is a non-competitive NMDAR antagonist used for treatment of Alzheimer’s disease. Two recent meta-analyses (42, 43) and one systematic review (44) have concluded that adjunctive memantine to antipsychotics is safe and significantly improves negative symptoms in chronic medicated patients with schizophrenia. One of the meta-analyses also indicated a potential beneficial effect on global levels of cognition of patients treated with adjunctive memantine compared to the placebo group (42).

Memantine has been theorized to ameliorate progression of negative symptoms by counteracting excitotoxicity correlated to high glutamate levels in early stages of psychosis (45), but this has not yet been investigated in initially antipsychotic-naïve patients.

**FIGURE 1:**
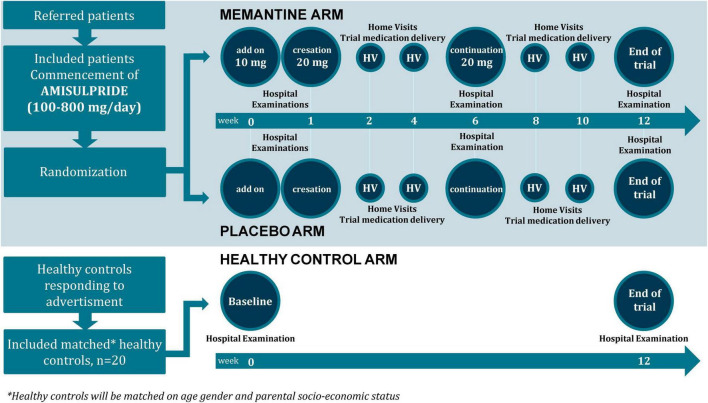
AMEND flow chart.

AMEND is an ongoing double-blinded clinical trial (Clinicaltrials.gov, ID: NCT04789915), where antipsychotic-naïve patients with first-episode psychosis will be randomly allocated to treatment with first-line antipsychotic compound with addition of memantine or placebo. Patients will be matched with HCs and all participants will undergo baseline and 12-weeks examinations (Figure 1).

We hypothesize that add-on memantine to amisulpride will be superior to amisulpride monotherapy in reducing negative symptoms, and that this effect will correlate with thalamic glutamate levels. Moreover, we anticipate that add-on memantine will restore regional white matter integrity and improve cognitive functioning.

## Methods and Analysis

### Study Design

Double-blinded randomized controlled trial (RCT). Phase I, known medicine (memantine) tested for new indication (treatment of negative symptoms in psychosis).

### Study Population

#### Patients

We will include antipsychotic-naïve people with schizophrenia spectrum first-episode psychosis. Patients will be recruited from psychiatric hospitals and outpatient psychiatric centers in the Capital Region and in Region Zealand of Denmark. Doctor or nursing staff will inform potentially relevant patients about the project, and if he/she accepts, contact the AMEND project medical doctor. Present State Examination (PSE), will be performed by the project medical doctor, to confirm a psychosis diagnosis. Included ICD-10 diagnosis are shown in Table 1. In conjunction, a global evaluation as per general clinical practice will be performed to assess whether antipsychotic treatment is indicated. Patients will be evaluated in cooperation with a senior consultant psychiatrist. Furthermore, a brief somatic and neurological examination, as well as screening for pregnancy and substance abuse, will be performed.

**TABLE 1:** In- and exclusion criteria in AMEND.

Patients
**Inclusion criteria**
+ Antipsychotic-naïve, first-episode psychosis
+ Fulfilling diagnostic criteria of schizophrenia, persistent delusional disorder, acute and transient psychotic disorders, schizoaffective disorder, other non-organic psychotic disorders and unspecified non-organic disorders (ICD-10: F20.x; F22.x; F23.x; F24.x; F25.x; F28; and F29)
+ Age: 18–45 years
+ Legally competent
**Exclusion criteria**
- Current substance dependence ICD-10 (F1x.2) or substance abuse in any period up to 3 months prior to referral (exception: tobacco/nicotine, F17.2)
- Treatment with antidepressants within 30 days
- Head injury with more than 5 minutes of unconsciousness
- Any coercive measure
- Metal implanted by operation
- Pacemaker
- Pregnancy (assessed by urine HCG)
- Female patients: Unwillingness to use safe contraception (Intra Uterine Device/System or hormonal contraceptives) during the study period.
- Severe physical illness
- Allergies to any of the inactive ingredients and film coat components

**Healthy controls (*N* = 20) matched on age and gender**
**Inclusion criteria**
+ No first-degree relative with known major psychiatric disorder (ICD-10: F1x; F2x; and F3x)
+ Age: 18-45 years
+ Legally competent
**Exclusion criteria**
- Lifetime substance abuse/dependence ICD-10 (F1x.1/F1x.2) (exception: tobacco/nicotine, F17.1/F17.2)
- Head injury with more than 5 minutes of unconsciousness
- Lifetime treatment with antidepressants
- Metal implanted by operation
- Pacemaker
- Pregnancy (assessed by urine HCG)
- Females: Unwillingness to use safe contraception (Intra Uterine Device/System or hormonal contraceptives) during the study period.
- Severe physical illness

Patients will receive no economical compensation for participation as remuneration is not allowed according to Danish regulations. The medication will be provided free of charge and transportations costs will be covered.

#### Healthy Controls

Twenty healthy controls (HC) matched 1:2 on age, sex, ethnicity and parental socioeconomic status will be recruited through online advertisement per website.^1^ Healthy controls will receive a taxable disadvantage allowance of DKK 1,000 for completion of the baseline visit and DKK 2,000 for the end of trial visit.

In- and exclusion criteria are shown in Table 1.

#### Trial Visits and Examinations

At baseline in the antipsychotic-naïve state as well as after 12 weeks of treatment, patients will undergo detailed examination program including: (1) clinical ratings and assessments, (2) neurocognitive testing, and (3) Magnetic Resonance Imaging (MRI) scans. Between week 0 and week 12 regular clinical examinations will be undertaken, see examination program Table 2.

**TABLE 2:** AMEND examination program.

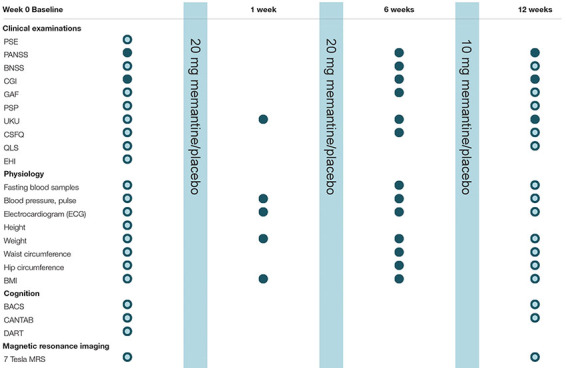

*
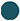
Patients 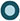
Patients and healthy controls*

***Clinical examinations:***

*Diagnosis/symptoms: Schedules for Clinical Assessment in Neuropsychiatry (SCAN), Positive And Negative Syndrome Scale (PANSS), Brief Negative Symptom Scale (BNSS), Calgary Depression Scale for Schizophrenia (CDSS).*

***Level of functioning:** Clinical Global Impression (CGI), Global Assessment of Functioning (GAF), Personal and Social Performance Scale (PSP).*

*Side effects/quality of life: Udvalg for Kliniske Undersøgelser (UKU) (measurement of side effects), Changes in Sexual Functioning Questionnaire (CSFQ), Quality of Life Scale (QLS).*

***Other:** Edinburgh Handedness Inventory (EHI). Total duration, clinical assessments: 2.5–3 h.*

*Physiology:*

Fasting blood samples include: Fasting plasma glucose, HbA1c, triglyceride, total cholesterol, LDL cholesterol, VLDL cholesterol, HDL cholesterol, prolactin, *se-amisupride, *se-memantine (*follow-up only).

*Body Mass Index (BMI).*

*Total duration, clinical assessments: 15 mins.*

***Cognition:***

*-Brief Assessment of Cognition in Schizophrenia (BACS): memory, working memory, processing speed, executive functions and verbal fluency (35–40 min).*

*-Cambridge Neuropsychological Test Automated Battery (CANTAB) (selected tests): Rapid Visual Information Processing (RVP) (attention), Spatial Working Memory (SWM) (working memory), Paired-Associates Learning (PAL) (visuo-spatial associative learning, i.e. ‘what, when and where’) (25-27 min).*

*-Danish Adult Reading Test (DART): premorbid IQ measure (5–6 min).*

*Total duration, cognition: 65-75 min.*

***Magnetic resonance spectroscopy (MRS):***

*Magnetic Resonance Spectroscopy measurements will performed Danish Research Centre for Magnetic Resonance (DRCMR) on a whole-body 7 T MR scanner (Achieva; Philips, Cleveland, OH, United States) using a 2-channel volume transmit head coil and 32-channel receiver array (Nova Medical, Inc, Burlington, MA, United States).*

*Voxel placement is planned in five a priori selected regions if interest (ROIs): thalamus, anterior cingulate cortex (ACC), hippocampus, dorsolateral prefrontal cortex (DLPFC), and basal ganglia (32–34).*

*MRS analyses will be performed in LCModel (Provencher, 1993) as also used in Bojesen et al. (9), Reid et al. (33), and Wang et al. (34).*

*Measurements of brain metabolites include: glutamate, glutamine, γ-aminobutyric acid (GABA), N-acetylaspartate, N-acetylaspartyl glutamate, and glutathione (33, 34).*

*Total duration, MRS: 60 min.*

#### Clinical Ratings

Symptom severity will be assessed by the Positive and Negative Syndrome Scale (PANSS) (46). Moreover, the primary clinical endpoint, PANSS negative symptoms, will be supplemented by the Brief Negative Symptom Scale (BNSS) (47). As descriptive and explorative endpoints, quality of life will be evaluated using the Quality of Life Scale (QLS) (48). The UKU side effect rating scale (49) and Changes in Sexual Functioning Questionnaire (CSFQ) (50) will be used to asses potential side-effects. Handedness will be assessed with Edinburgh Handedness Inventory (EHI) (51). Level of functioning will be evaluated with the Personal and Social Performance Scale (PSP) (52), and Global Assessment of Functioning (GAF) (53). The Clinical Global Impression (CGI) (54) will be used to assess the patients’ overall illness severity.

Based on previous studies in similar patient populations we expect the mean PANSS negative symptom score to reside around 20, eg. (9, 55) and in order to enhance generalizability to this patient population, we did not set a minimal level of symptoms.

In order to retain comparability to other studies, e.g.,(42–44) we opted for PANSS. To address the controversy regarding of PANSS negative symptoms, we employ BNSS as secondary endpoint. We recently validated BNSS for use in Danish settings (56, 57).

#### Cognitive Testing

Specific cognitive functions will be examined using selected tasks from The Cambridge Neuropsychological Test Automated Battery (CANTAB) (58–60) and The Brief Assessment of Cognition in Schizophrenia (BACS) (61, 62). Both batteries are well validated and have previously been applied in patients with schizophrenia, e.g., (26, 63–68).

From CANTAB we will include Spatial Working Memory (SWM) to assess visual working memory, Paired Associated Learning (PAL) to assess visual learning, and Rapid Visual Information Processing (RVP) to assess sustained attention. BACS will be used to assess verbal learning, verbal working memory, processing speed, planning and verbal fluency. Finally, premorbid intelligence (IQ) will be estimated using the Danish version of the National Adult Reading Test (DART) (69) (adapted from the National Adult Reading Test, NART) (70).

### Magnetic Resonance Imaging and Magnetic Resonance Spectroscopy

Magnetic resonance imaging scans will be performed at the Danish Research Centre for Magnetic Resonance (DRCMR), Copenhagen University Hospital Amager and Hvidovre, on a whole-body 7 Tesla (7T) MR scanner (Achieva; Philips, Cleveland, OH, United States) using a 2-channel volume transmit head-coil and 32-channel receiver array (Nova Medical, Inc., Burlington, MA, United States). MRS will be used to assess glutamate levels pre – and post treatment in five preselected regions of interest: anterior cingulate cortex (ACC), dorsolateral prefrontal cortex (DLPFC), thalamus, hippocampus, and the basal ganglia. In addition, T1- and T2 weighted structural images and diffusion sensitive sequences will be performed to explore cortical and subcortical brain structures, and the integrity of white matter tracts. Quantitative Susceptibility Mapping (QSM) will be used to estimate regional iron accumulation, and measurement of the neuromelanin (NM) concentration will be performed in substantia nigra (SN) as a proxy for structural and functional integrity of dopaminergic cells (36).

Our group has established a robust 7T protocol for glutamate MRS at our 7T MR system and validated the protocol in a cohort on healthy individuals in a study on normal cognitive aging (71, 72).

We will apply this optimized MR protocol in the AMEND study on a Philips 7T whole body MR scanner (Philips) using a dual transmit coil and a 32-channel receive head coil (NovaMedical). We will use a sLASER sequence (73) with a TR/TE = 3700/32 ms, bandwidth = 4 kHz, data points = 2048) with prospective frequency and motion correction.

For structural MRI, we will use a tailored acquisition protocol which we have successfully applied to map the SN and LC at high resolution and signal-to-noise ratio in a cohort of patients with Parkinson’s disease and age-matched controls. The neuroimaging results regarding the Locus coeruleus have been recently published (74). In addition to locus coeruleus mapping, this structural 7T MR protocol also provides exquisite high-resolution maps of the substantia nigra (unpublished data). In short, the structural MRI protocol includes a T1-weighted(T1w), high-resolution (1-mm isotropic) and a magnetization transfer weighted, three-dimensional, high-resolution (voxel size, 0.4 × 0.4 × 1.0 mm) ultra-fast gradient echo sequence (echo time/repetition time = 4.1/8.1 ms, flip angle = 7 degrees, two averages). Magnetization transfer saturation will be achieved by applying 16 block-shaped pre-pulses at a frequency offset of 2 kHz (flip angle = 278 degrees, duration = 10 ms). We will also acquire T1-weighted images with identical acquisition parameters for co-registration purposes, but with a zero-degree flip angle for the off-resonance pre-pulse.

### Intervention

#### Amisulpride

Tablet *amisulpride* is a second-generation antipsychotic and a first-line, generic drug against psychosis and schizophrenia in Denmark (75). Amisulpride primarily binds to dopamine D2 and D3 receptors, but also has affinity for serotonin 5-HT7 receptors (76). In AMEND, amisulpride is used as “tool compound” because it is the most selective licensed antidopaminergic compound to treat psychosis. All patients will be treated with tablet amisulpride with initial dose 50–100 mg/day. Doses will be slowly augmented according to effect and side-effects to a maximum of 800 mg/day.

#### Memantine vs. Placebo

Tablet *memantine* is approved for treatment of Alzheimer’s Disease (AD) showing benefits on the main domains of AD i.e.,. cognition, function, behavior, and clinical global change (77). Memantine acts through uncompetitive, open-channel NMDAR antagonism, and has minimal activity for GABAergic, dopaminergic, adrenergic, histaminergic, and glycinergic receptors.

Patients will be blindly randomized to treatment with either memantine or placebo. Treatment will be initiated at 10 mg/day for 1 week, and from the second week the dose will be increased to 20 mg/day until end of trial. Tablets will be identical, provided in 10 mg or 20 mg tablets, and labeled by the Hospital Pharmacy (Region Hovedstadens Apotek) according to GCP-guidelines; marked with trial reference number, trial subject reference, dosage, administration route, storage and expiration date.

### Randomization

The Hospital Pharmacy will be responsible for randomization, which will be conducted 1:1. Randomization will be stratified by PANSS negative sub-score, categorizing patients in either [Low PANSS negative baseline <20] or [High PANSS negative baseline ≥20].

The randomization will use permuted blocs in sizes of 4,6 and 8 to ensure an even distribution among the two groups. The number of patients in each group will not be restricted. In need of unblinding of a patient’s treatment, the pharmacy can be contacted at any time.

Intolerable side-effects or clinical worsening (defined as a 20% increase in PANSS positive subscale score compared to baseline PANSS positive score) will result in discontinuation of study treatment and study exclusion. Patients will be consulted with the senior consultant psychiatrist associated to the research project and further treatment will be initiated according to general treatment guidelines respecting the patients’ autonomy and requests.

### Compliance

All tablets are provided to patients free of charge. Tablets will be administered with regular time intervals by a research nurse at the hospital or at home visits (Figure 1). Compliance will be assessed biweekly by phone-call or at clinical visits, where patients are asked how many days, if any, medication was not taken. Serum-amisulpride and serum-memantine will be measured by blood samples taken at the research department at 6 weeks and at end of treatment.

### Data Analysis

#### Primary Endpoint

The primary endpoint will be reduction in negative symptoms as measured with the negative symptom score from the Positive and Negative Syndrome Scale (PANSS) (78). Reduction in negative symptoms after 12 weeks of treatment: [PANSS negative baseline–PANSS negative at week 12]. PANSS negative assessments will be supplemented by Brief Negative Symptom Scale (BNSS) scores (57).

#### Secondary Endpoints

Secondary endpoints include changes in cognition (in particular working memory, learning and processing speed, and sustained attention), PANSS positive and total-scores, other clinical measures, level of functioning, adverse effects, and glutamate levels in the five *a priori* selected regions of interest mentioned above. Additionally, dopamine function in substantia nigra will be explored using NM-MRI techniques (37).

#### Exploratory Endpoints

Exploratory endpoints include associations between clinical data, quality of life, brain metabolites in other regions, e.g., ACC (33, 34), structural measures (e.g., hippocampus subfields) (35), diffusion data, and basal ganglia quantification (Table 2). Interactions between baseline brain metabolite levels and brain structure and white matter integrity in patients and HC will also be investigated.

### Planned Statistical Analyses

Homogeneity of variance between the two groups will be assessed using Levene’s test. Parametric data will be evaluated using parametric testing. Non-normally distributed data or data that exhibit unequal variances will be tested using non-parametric tests as appropriate. Fisher’s or χ2 exact tests will be used for group comparisons between categorical data. The Benjamini-Hochberg procedure will be used to control the type I error rate.

Data analyses on the primary outcome and most of the secondary outcomes will be performed before un-blinding and according to the per-protocol principle. Analyses of adverse events will be performed according to the intention-to-treat principle. Demographic variables and clinical characteristics will be reported in frequency for categorical data, and in mean values (with standard deviations and range) for normally distributed, continuous variables. Group comparisons for demographic data will be performed using independent *t*-tests for continuous variables and Chi-square tests for nominal and ordinal variables. Primary and secondary outcomes will be tested using repeated measures (rm)ANOVA.

### Power Calculation

The power calculation is based on the primary outcome. We used the most representative clinical study we could identify and attempted to modify to the current setting. An 8-week RCT using risperidone plus memantine in stable schizophrenia patients (*N* = 40) reported a reduction in PANSS negative of −2.8 (SD 1.6) with memantine compared to −0.8 (SD 0.9) with placebo (79). Assuming a similar response, we expect add-on memantine to induce −2 ± 2 point more reduction in PANSS negative compared to amisulpride monotherapy. An *a priori* Student *t*-test (two-sided) with significance level α of 0.05 and a desired power (1-α) of 80%, shows that 16 completed patients in each arm is needed.

To increase power of secondary analyses, enrollment will continue until 18 participants in each arm have completed examinations. Moreover, AMEND will be extended to 12 weeks based on the median trial duration in the meta-analysis of add-on memantine [12 weeks (mean 11.5 weeks)] (42). Based on our center’s 20 years of experience with recruitment and treatment of antipsychotic-naïve patients, we expect an inclusion rate of 2 patients per month and an attrition rate of 25%. Thus, recruitment of 46 patients will take approximately 2 years.

## Discussion

By rational combination of two licensed, off-patent drugs, amisulpride and memantine, AMEND aims to optimize treatment of psychosis, especially the accompanying negative and cognitive symptoms, alongside with unraveling the signature of memantine response. If successful, AMEND will provide pivotal neurobiological evidence for future stratification of patients with first-episode psychosis before initial antipsychotic treatment.

There are few potential risks related to participating in the AMEND study. Side effects of memantine are relatively mild compared to antipsychotic medication. In previous memantine add-on RCTs in schizophrenia patients, the reported side effects did not differ from placebo (42) and chronic patients treated with memantine as add-on treatment had an improvement compared to placebo (79), why we assume that the added treatment also will improve patients with first-episode psychosis. The participants will have the advantages of a thorough examination program with more detailed psychopathological and neuropsychological testing than normally provided.

Magnetic resonance imaging procedures have not been associated with any health risk, however, the examination might cause discomfort for people with claustrophobia and anxiety. Participants will be followed carefully during the project and any potential discomfort will be attempted minimized by research staff. Great care will be taken by the research group to ensure that all examinations are carried out as smoothly and efficiently as possible, including breaks when needed. Scanning will be discontinued in cases of discomfort or anxiety, and communication between staff and participants will be possible during the whole scan.

Participants with head/neck tattoos or permanent make-up might experience warming and soreness of the painted area, and scanning will be interrupted should these symptoms arise. Incidental findings on MRI scans might lead to worrying and further clinical work-up. Blood samplings might cause discomfort for some people but will be taken by trained personal and under hygienic circumstances.

Participants can withdraw from the study at any time without explanation, and without any consequences for the following treatment.

Regarding the statistics, our power calculation and number of participants are not sized for all exploratory analyses, but our RCT design and previous pilot data, justify the rationale for conducting these tests. Admittedly, a mean 2-point reduction in PANSS negative symptoms may only represent a modest clinical improvement for the individual patient participating in this study. However, the RCT design in combination with 7T MRS data will provide strong neuroscientific evidence on how NMDARs may mediate effects on unresolved symptom domains. If successful, the combined clinical and neurobiological insights from AMEND will provide an important steppingstone, which will have major impact for further research, for stakeholders in the pharmaceutical industry, and for future patients with psychosis.

With the ambitious goal of moving clinical psychiatry toward pre-treatment stratification based on pathophysiological markers, AMEND will provide evidence for rational drug repurposing to optimize treatment outcome for patients with psychosis.

## Ethics and Dissemination

The trial has been approved by the Danish Health and Medicines Authority, the National Committee on Health Research Ethics, Danish National Committee on Biomedical Research, and the Danish Data Protection Agency. Trial participation presupposes oral and written informed consent of all participants. Memantine will be used off-label, and tolerability and clinical symptoms will be monitored closely. Personal integrity and privacy concerning participants will be protected by the Danish Law of Health. The study will be conducted in accordance with the Helsinki Declaration II and monitored according to Good Clinical Practice principles (ICH-GCP). AMEND is registered at EudraCT nr: 2021-001061-20 and ClinicalTrials.gov (NCT04789915). The trial can be subjected to quality audit. Research data and results (positive and negative), will be presented at national and international scientific meetings and conferences. Articles will be submitted to peer-reviewed journals. Authorships will adhere to the International Committee of Medical Journal Editors statement (ICMJE).^2^

## Ethics Statement

The studies involving human participants were reviewed and approved by Danish Health and Medicines Authority, the National Committee on Health Research Ethics, Danish National Committee on Biomedical Research, and the Danish Data Protection Agency. The patients/participants provided their written informed consent to participate in this study.

## Author Contributions

BE conceived the study. KB, MN, BF, BG, and HS contributed to the study design was adjusted in discussions. AM, CL, VB, HL, DS, MS, AL, WS, and CP refined the clinical- and neuropsychiatric examination programs with respect to their respective fields of expertise. KS, OB, and BE drafted the manuscript. All authors approved the submitted version of the manuscript.

## Conflict of Interest

BE was part of the Advisory Board of Eli Lilly Denmark A/S, Janssen-Cilag, Lundbeck Pharma A/S, and Takeda Pharmaceutical Company Ltd; and has received lecture fees from Bristol-Myers Squibb, Boehringer Ingelheim, Otsuka Pharma Scandinavia AB, Eli Lilly Company, and Lundbeck Pharma A/S. HS has received honoraria as speaker from Sanofi Genzyme, Denmark and Novartis, Denmark, as consultant from Sanofi Genzyme, Denmark, and Lundbeck AS, Denmark, and as editor-in-chief (Neuroimage Clinical) and senior editor (NeuroImage) from Elsevier Publishers, Amsterdam, The Netherlands. He has received royalties as book editor from Springer Publishers, Stuttgart, Germany and from Gyldendal Publishers, Copenhagen, Denmark. The remaining authors declare that the research was conducted in the absence of any commercial or financial relationships that could be construed as a potential conflict of interest.

## Publisher’s Note

All claims expressed in this article are solely those of the authors and do not necessarily represent those of their affiliated organizations, or those of the publisher, the editors and the reviewers. Any product that may be evaluated in this article, or claim that may be made by its manufacturer, is not guaranteed or endorsed by the publisher.

## Footnotes

1
www.forsoegsperson.dk

2
http://www.icmje.org/recommendations/
